# Dr. Halliday, on Epilepsy

**Published:** 1808-04

**Authors:** Andrew Halliday

**Affiliations:** Blandford


### *To the Editors of the Medical and Phyſical Journal.*

Gentlemen,

On the 28th of December, 1807, I was requested by Corporal Good, of His Majesty's 13th Regiment of Light Dragoons, to visit *Jane Good*, his daughter, a girl about 5 years of age. I accordingly went with him to his quarters, and found the child in question quite recovered from a very severe fit, which was described to me by her mother in nearly the following terms. At the approach of the fit, she cries out most violently, as if suffering the most excruciating pain, and which continues for two or three minutes. The muscles of the trachea and extremities become then affected with violent spasms. The thighs at times are drawn up upon the abdomen; while the head and shoulders are drawn backwards, and the breast pushed out; at other times the head is pulled forward, and the chin is pressed upon the upper part of the chest. The thighs remain stretched out, and the legs only are contracted backwards. This state seldom continues longer than four or five minutes, and generally goes off with twitchings of the muscles. She then lies calmly in whatever position she is laid, but seems to breathe with difficulty, and there is a continued motion of the lower jaw; and when the fit is about to leave her, she begins to moan very much, and frequently sighs. When I saw her first, she had just recovered from one of these paroxysms, and was sitting upon the bed; there was not only a constant motion of the lower jaw, but also of the upper extremities; she was continually tossing her arms about, and also twisting and turning her head. During the paroxysm, a red-hot poker had fallen from the grate upon her left leg and burnt it very severely, yet she had expressed no symptoms of feeling pain. When I began to ask her some questions, I was informed by her mother that she did not articulate properly, and at the same time, she gave me the following history of the complaint. When 2 years of age, the child then stout and very healthy, accidentally pulled over a large bason full of hot water, which falling upon her breast, scalded her in several parts very severely, particularly about the scrobiculus cordis, and on the left side of the inferior part of the sternum; it was near three months before she recovered from this accident; and long after the sores were healed, she continued very weak and unhealthy. The first symptoms of her pre-

 sent sent complaint appeared about two months after the above-mentioned accident, but they were very slight, and did not recur for some months. They have gradually, however, become more severe, and their recurrence has become more and more frequent during the last two years; and at present she is seldom four hours free from them, night or day. She seems conscious of their approach, as she begins to scream and express terror for some minutes before the spasmodic actions, which I have already described, take place. She knows what is said to her when free from the fits, yet she can articulate but very few words. Her appetite is most voracious and depraved; she is fonder of coal-cinders, pieces of clay and chalk, than of wholesome food; and if allowed, she would sit constantly close under the grate, picking up the cinders and eating them. When kept from the fire, she seems to feel the cold very much.

Her parents could assign no cause for her complaint, except the severe accident mentioned. I was told that she had been for some time under the care of a surgeon at Sandwich, last summer, without receiving any apparent benefit.

Upon an attentive consideration of this case, it occurred to me that purgatives were likely to be of service; and from my intimate acquaintance with the practice of the justly celebrated physician, Dr. Hamilton of Edinburgh, I entered upon the treatment with great confidence, and did not hesitate to promise success to the parents of the girl, if they would faithfully and implicitly follow my directions. I confess that I had my fears, lest there should be some organic disease, yet the pulse, though rather slow, was regular. The bowels I was told were very irregular, but generally costive; I felt the abdomen very tumid; and notwithstanding the feebleness and emaciated state of the patient, I felt convinced that no time was to be lost; I therefore ordered the following purgative, viz.

℞. Submuriatis hydrarg. gr. vi. Pulveris jalapæ ℈j.—Conservæ rosa. q.s. ut fiat bolus cras primo mane sumendus.

Dec. 29.—Bolus was taken about six o'clock this morning. As yet no stool. Had four fits in the course of the night, and two this forenoon; as usual, very severe. Has drank a considerable quantity of water gruel this morning, and is still calling for more.

℞. Infus. sennæ ℥viij. sumat unciam omni semihora donec plene responderit alvus.

Dec. 30.—Has had several motions, scanty, fluid, of a greenish colour, and highly fœtid. Has had five very severe fits since yesterday. Repetat.

Repetat. bolus cras mane et horis quatuor (elapsis, post bolum sumptum.) Repetat. etiam infusum sennæ ut heri.

Dec. 31.—The bolus and four ounces of the infusion of senna have been taken. They have produced two stools similar to those of yesterday; and with the last motion, a lumbricus seven inches in length, and of considerable thickness, was voided. Capiat quid reliqui sit infusi sennæ.

℞. Submuriatis hydrarg. gr. xij. Pulveris radicis rhei ℈j. Fusci. sacchari, cochlear. medium. M. fiat electuarium quod capiat cras vice bol.

Jan. 1, 1808.—Had one motion last night, and has had two this morning; since taking the electuary, has voided another lumbricus. The fits have been more severe during last night and this morning, than they were ever observed to be since their commencement. The fæces are still scanty and fluid. The abdomen feels hard, and is still prominent.

Jan. 3.—Has had two motions since yesterday, and one on the evening of the 1st. The last is very copious, consisting chiefly of hardened scybala. Two lumbrici have been voided with the last motion. She had no fits during the whole of yesterday; but they have been both as frequent and severe during the night.

Repetat. electuarium ut antea præscripti cras mane et vespere versus habeat infus, sennæ ℥ij. Habeat juris bovilis ℔iij. vel iv. in dies.

Jan. 4.—Had only one fit in the course of yesterday; yet they were very frequent during the night; but her mother says they were less severe than they have been for some time past. Has had copious dark coloured alvine evacuation. Her appetite is less voracious.

Jan. 6.—Had two motions in the course of yesterday, and has voided another very large lumbricus, and a great quantity of hardened scybala. The fits do not recur during the day-time, but were very frequent on the night of the 4th and last night; but their duration is much shorter, and they have been much less severe than usual.

Repetat. electuarium ut antea, et repetat. etiam infusum sennæ.

---

From this time to the 20th, I continued the exhibition of calomel and rhubarb, and the senna occasionally; never intermitting more than one day. The quantity of feculent matter which she passed during that period is beyond conception. Her appetite began to flag about the 14th, and on the 16th her mother informed me that she had not had a fit for twenty-four hours. On the 17th she had one very severe fit, but she remained free from them again till the 20th, when she had one which did not continue above ten minutes. She voided three lumbrici between the 6th and 20th.

On the 21st I find the following entry in my note book. Appetite nearly natural; one scanty stool without scybala; no fit since yesterday.

℞. Mas. pilul. ex aloe & colocynth ʒj. Divide in pilulas xij. sumat ij. 6ta quaque hora.

Jan. 23.—The pills have been taken and have produced two motions. The last formed, and of natural appearance, but fœtid. No fit since the 20th.

℞. Mas. pilul. ex aloe & colocynth ʒiij. divid. in pilulas xxxvj. Sumat ij. vel iij. pro re nata.

---

From this time till the 2d of February, I had not an opportunity of seeing my patient; she had two or three very slight fits during the week. She had taken the pills so as to procure, at least, one motion daily; her abdomen was soft and natural; she continued still fond of eating cinders and pieces of chalk. She can articulate but few words, and is, to all appearance, a complete idiot; yet she has improved much in flesh and strength during the last ten days. I ordered another box of pills, and directed them to be given so as to secure a regular alvine discharge; and desiring Corporal Good to call upon me again if she did not continue to mend, I took my leave.

To day, however, I was passing the house, and called to ask after my little patient. I found her sitting by the fire, very much improved in her looks; and learnt from her mother that she had remained free from the fits for the last fortnight; that her appetite was becoming more natural, both with regard to the quantity and nature of her food.

Thus far, Gentlemen, the purgatives have fully answered my expectations. The child appears to be cured of her fits, but I am afraid she will remain an idiot while she lives. You must have remarked the very large doses of medicine that were necessary to move her bowels, and also the length of time which elapsed before the bowels could be said to be properly moved. For I conceive, that notwithstanding the great quantity of calomel which she took, she had no proper motion till the 3d of January.—The large doses of medicine which were necessary, may be accounted for, perhaps, from the state of the sensorium; and the difficulty which there was in moving the bowels, bowels, was, no doubt, owing to the great accumulation which had taken place.

Though the fits are removed at present, I fear they will be apt to return, unless great care is taken to keep her bowels open for some considerable time, until the predisposition from habit is overcome, and the bowels are restored to their natural tone; but if this is attended to, I am certain the cure will be complete. This Case then, I would say, tends to corroborate the very valuable Observations of Dr. James Hamilton, but indeed those Observations stand not in need of any such testimony; for Dr. Hamilton has proved every position, which he has advanced by facts that never can be controverted. The novelty, the simplicity, and the efficacy of Dr. Hamilton's practice, attracted much notice on the first appearance of his invaluable work; and as the Doctor did not venture to give his discoveries to the world till experience had most fully confirmed them, he was able to speak with certainty; and I will venture to affirm, that if purgatives have failed in any instance to produce the effects which Dr. Hamilton's Observations have so incontestibly proved them capable of producing, that that failure is to be attributed more to the prescriber than to the medicines prescribed.

I have often heard it argued, by those who were unwilling to give too much credit to Dr. Hamilton, as was generally allowed, that though no doubt the cases which he had related, seemed to prove the good effects of purgatives; yet that many of those cases; for example, his cases of Typhus Fever, were so trifling, that any other remedy would have done as well as purgatives. And, moreover, it has been often hinted, that though this practice may do very well in the north, and in the Edinburgh Royal Infirmary, yet that it is by no means calculated for the delicate constitutions of this country. I shall only say, Gentlemen, that those who have witnessed Dr. H's practice, have been fully convinced of the good effects of purgatives in severe as well as slight cases of fever; and, indeed, had the Doctor felt any anxiety about this, he might have filled the 2d number of his Appendix with cases more severe than any he has given. With regard to the second hint, I can add my testimony to that of Dr. Morgan of Dover.* I have prescribed purgatives in various diseases since my residence in England, and have found their effects uni-

 formly

* See Einburgh Medical and Surgical Journal, April 1, 180[illegible]

formly the same as in the north. While I resided at Halesworth in Suffolk, I attended *Robert White* of Walpole, with Mr. Walker, Surgeon, in one of the worst cases of typhus I ever saw. The disease was speedily subdued by purgatives. The *Bolus Jalapæ Compositus* had the same good effect in Suffolk, as in the Royal Infirmary of Edinburgh.

I am, &c.

ANDREW HALLIDAY, M. D.

*Blandford, February* 22, 1808.

